# Is home where the heat is? comparing residence-based with mobility-based measures of heat exposure in San Diego, California

**DOI:** 10.1038/s41370-024-00715-5

**Published:** 2024-09-11

**Authors:** Michael D. Garber, Anaïs Teyton, Marta M. Jankowska, Gabriel Carrasco-Escobar, David Rojas-Rueda, Antony Barja-Ingaruca, Tarik Benmarhnia

**Affiliations:** 1 Scripps Institution of Oceanography, University of California, San Diego, San Diego, California USA; 2 Department of Environmental and Radiological Health Sciences, Colorado State University, Fort Collins, Colorado USA; 3 School of Public Health, San Diego State University, San Diego, California USA; 4 Herbert Wertheim School of Public Health and Human Longevity Science, University of California, San Diego, California USA; 5 Population Sciences, Beckman Research Institute, City of Hope, Duarte, California USA; 6 Universidad Peruana Cayetano Heredia, San Martín de Porres, Lima, Peru; 7 Irset Institut de Recherche en Santé, Environnement et Travail, UMR-S 1085, Inserm, University of Rennes, EHESP, Rennes, France

**Keywords:** Urban heat, Activity space, GPS, Exposure assessment.

## Abstract

**Background:**

Heat can vary spatially within an urban area. Individual-level heat exposure may thus depend on an individual’s day-to-day travel patterns (also called mobility patterns or activity space), yet heat exposure is commonly measured based on place of residence.

**Objective:**

In this study, we compared measures assessing exposure to two heat indicators using place of residence with those defined considering participants’ day-to-day mobility patterns.

**Methods:**

Participants (*n* = 599; aged 35-80 years old [mean =59 years]) from San Diego County, California wore a GPS device to measure their day-to-day travel over 14-day intervals between 2014-10-17 and 2017-10-06. We measured exposure to two heat indicators (land-surface temperature [LST] and air temperature) using an approach considering their mobility patterns and an approach considering only their place of residence. We compared participant mean and maximum exposure values from each method for each indicator.

**Results:**

The overall mobility-based mean LST exposure (34.7 °C) was almost equivalent to the corresponding residence-based mean (34.8 °C; mean difference in means = −0.09 °C). Similarly, the mean difference between the overall mobility-based mean air temperature exposure (19.2 °C) and the corresponding residence-based mean (19.2 °C) was negligible (−0.02 °C). Meaningful differences emerged, however, when comparing maximums, particularly for LST. The mean mobility-based maximum LST was 40.3 °C compared with a mean residence-based maximum of 35.8 °C, a difference of 4.51 °C. The difference in maximums was considerably smaller for air temperature (mean = 0.40 °C; SD = 1.41 °C) but nevertheless greater than the corresponding difference in means.

**Impact:**

As the climate warms, assessment of heat exposure both at and away from home is important for understanding its health impacts. We compared two approaches to estimate exposure to two heat measures (land surface temperature and air temperature). The first approach only considered exposure at home, and the second considered day-to-day travel. Considering the average exposure estimated by each approach, the results were almost identical. Considering the maximum exposure experienced (specific definition in text), the differences between the two approaches were more considerable, especially for land surface temperature.

## Introduction

As the climate warms globally, it is increasingly important to address exposure to extreme urban heat and its health-related risks [1], given that most of the population lives in urban areas [2]. This need was underscored by the extreme heat of the summer of 2023, which was the hottest on record [3]. Globally, about 500,000 deaths annually have been estimated to be attributable to excess heat [4]. Features of the built and natural urban environment contribute to the variation of heat between urban areas and their surrounding rural areas (often called the urban heat island effect) and within urban areas (intra-urban heat) [5]. Impervious surfaces (e.g., asphalt, concrete) and buildings absorb heat [6], while trees, vegetation, and exposed soil which can have cooling effects [2]. In U.S. settings, intra-urban variation in heat commonly results in inequitably high heat exposure in socially vulnerable areas of the city, such as those with high poverty or a high proportion of non-white residents [7, 8].

Environmental conditions affecting human thermal comfort include air temperature, wind speed, mean radiant temperature, and humidity, but measurement of these conditions at a high spatial resolution throughout the full extent of an urban area is rarely feasible. As a result, research on intraurban heat commonly uses remote-sensing-derived land-use indicators with high spatial resolution and global coverage such as land-surface temperature to serve as a proxy for personal heat exposure [5, 9, 10]. Gridded data on air temperature are also available for the U.S. [11], but at a coarser spatial resolution.

In epidemiologic studies, heat indicators obtained from gridded data are commonly linked to individuals based on residential location [12]. However, given the noted intraurban variation in heat [7], heat exposure may depend not only on residential location but also on the locations a person visits throughout their routine day-to-day travel (also called “activity space”) [13, 14]. An activity-space-based, dynamic, or otherwise non-residential approach to measure exposure is now widely used across several health-relevant exposures [13, 15]. For example, the paradigm has been used to measure exposure to alcohol and tobacco retail outlets [16, 17], food-outlet accessibility [18], physical activity and active travel [19], and air pollution [20, 21].

An activity-space-based approach may also be well-suited for assessment of heat exposure in epidemiologic studies, depending on the research question. For example, acute exposure to extreme heat can affect the transient risk of acute myocardial infarction or heat stroke [22–24]. If such extreme heat existed within an individual’s activity space but not near their home, exposure assessment considering their place of residence alone would lead to exposure misclassification. On the other hand, cumulative heat exposure is also relevant for health. For example, chronic heat exposure can lead to dehydration, consequently causing strain on the cardiovascular and renal systems [1]. Notably, residential nighttime heat exposure is a particularly important time window, as the urban heat island effect is most pronounced at night when individuals are usually home [25, 26].

Given that heat exposure both at and away from home is important for health, it is important to understand the extent to which results from heat-exposure assessment methods that consider residence alone differ from those that also consider day-to-day travel patterns. One study in Bangladesh compared residence-based with mobility-based assessment of land-surface temperature exposure and found that a residence-based approach alone underestimated heat exposure in some groups [27]. There is a need for additional research comparing the two approaches in U.S. settings. Therefore, our objective is to compare mobility-based and residence-based measures of exposure to two indicators of the outdoor heat environment (land surface temperature and air temperature) in a study population of Southern California residents. We stratify the comparison of measurement methods by daily distance travelled and socio-demographic characteristics to inform future research on heat exposure in socially vulnerable groups who may be at higher risk of heat exposure and its health consequences.

## Methods

### Study population and setting

We assessed heat exposure in the Community of Mines Study, an observational study with enrollment between 2014 and 2017 in San Diego County, California. San Diego County has a population of about 3.3 million per the 2020 census [28]. The urban area of San Diego County has an arid Mediterranean climate with average daytime high temperatures in the mid 70 degrees Fahrenheit in the summer and in the mid 60 degrees Fahrenheit in the winter [29]. Annual precipitation totals less than 12 inches, with most rainfall occurring during the cooler months. The county includes mountains and sparsely populated areas in its eastern region, which receives snowfall [28].

The Community of Mines protocol is available elsewhere [30], and aspects of the study have been described in other research [20, 31]. Briefly, 602 adults aged 35-80 years old (mean age=59 years) who had lived for at least 6 months in a selected census block group completed the study. The study population is comprised of 56% women and is ethnically (42% Hispanic/Latino) and socioeconomically diverse (21% income $30,000 or less; 60% $55,000 or more) [20, 31]. Study ethics approval was obtained from UCSD IRB protocol #140510. Signed informed consent was obtained from all participants who enrolled in the study.

### Participant GPS measures

Participants were instructed to wear Qstarz GPS devices (Qstarz International Co. Ltd, Taipei, Taiwan) during waking hours to measure their movement [30, 31]. The GPS observations, which we also call pings, each had a latitude value, a longitude value, and a time stamp. GPS data were processed, cleaned, and aggregated to the minute level, as detailed in the appendix of Jankowska et al. [31].

Of the 602 participants, 599 participants wore devices for at least one valid day, where a valid day is defined as having at least 10 h of wear time [31]. We assume pings that occurred one minute apart occurred while the GPS was being worn. Participants wore GPS devices on average for 13.8 days and for 13.3 h per day (median=13.8 h per day) at various two-week intervals between 2014-10-17 and 2017-10-06. The average GPS follow-up of about 14 days corresponds to the minimum number of days recommended to measure activity spaces [14]. Among those pings that did not occur one minute apart, the per-person mean duration between pings was 10.7 h (median=10.2 h), and there were on average 14.1 such pings per participant. We assume these pings with an interval longer than one minute represent non-wear time between going to bed and waking the next day.

We created activity paths [16] with each participant’s GPS data, ordering each participant’s GPS pings in time. We excluded points with as-the-crow-flies speeds exceeding 100 miles per hour (0.04% of person-time recorded) from that point to the next point in time. Upon inspecting these segments visually, they appeared to either represent flights, or implausible or erroneous GPS bouncing. We also excluded points outside San Diego County (1.1% of total person-time).

To descriptively characterize the activity space of participants, we measured their average distance traveled per day, where distance traveled is the cumulative distance between their GPS pings for that day. We also calculated their average daily time spent at home (home defined below) within a 200 m buffer of their home, including non-wear time.

### Heat indicators and data sources

We used two gridded measures of heat: land surface temperature (LST) and air temperature (Table 1). LST is defined as “how hot the surface of the Earth would feel to the touch in a particular location” [32]. We downloaded LST data from Landsat 8-9 Collection 2 Level 2 Band 10 ST [33] from the U.S. Geological Survey’s EarthExplorer data portal (https://earthexplorer.usgs.gov; accessed April 29^th^, 2024). The data are available every 15 days at a spatial resolution of 30 meters. We examined 69 images over San Diego County (Path 40, Row 37) between 2014-09-30 and 2017-10-08. We removed images with more than 3% cloud cover, resulting in a total of 35 images. Specific image identifiers and dates appear in eTable 1.

**Table 1:** Description of heat indicators.

						Distribution of measure over area in which participants traveled in San Diego County, California^a^			
Heat indicator	Data source	Spatial resolution	Temporal resolution	*N*, images	Dates of first and last image	Mean of daily means	Mean of daily SD	Max. of daily means	Min. of daily means
Land Surface Temperature (°C)	Landsat 8-9 Collection 2 Level 2 Band 10 ST, United States Geological Survey [33]. Downloaded from USGS EarthExplorer (https://earthexplorer.usgs.gov)	30 m	Every 15 days	35	2014-09-30—2017-10-08	31	8.36	46	13
Maximum daily air temperature (°C)	gridMET [11]	4 km	Daily	1054	2014-10-17—2017-10-06	25	3.75	41	9
Minimum daily air temperature (°C)	gridMET [11]	4 km	Daily	1054	2014-10-17—2017-10-06	12	3.02	22	0

^a^within a 200 m buffer of the area traveled by all study participants during the study period (regardless of time elapsed).

*SD* Standard deviation, *Max.* maximum, *Min.* minimum, *°C* Degrees Celsius.

The important advantage of Landsat-measured LST for this research is its high spatial resolution (30 m), which is fine enough to measure a wide street [34]. LST has limitations, however, as a direct measure of human heat exposure [10]. We thus also gathered gridded data on air temperature from temperature from gridMET [11], which may more closely reflect human thermal comfort than LST [5, 10]. The gridMET air-temperature data are also available at a higher temporal resolution than LST (daily) and allow consideration of intra-diurnal variation in heat, as the data include both daily maximum and minimum air temperature. The gridMET air-temperature has a coarser spatial resolution than the Landsat LST data, however. Its spatial resolution of 4 km corresponds roughly to a neighborhood-scale air-temperature measure [35]. We gathered 1054 days of gridMET data for both maximum and minimum air temperature in San Diego County.

**Fig. 1:**
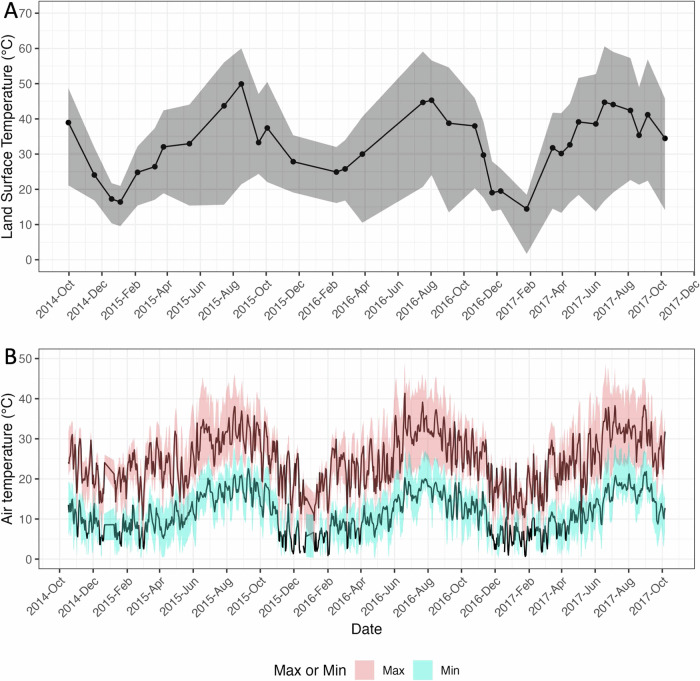
Distribution of the two heat indicators over the area traveled by all study participants over the study period. The points in panel **A** depict the median land surface temperature over the area traveled by study participants on each day with imagery. Lines between points in panel **A** are imputed values to facilitate visualization. The dark lines in Panel **B** depict the daily median maximum air temperature and daily median minimum air temperature over the area traveled by study participants. The shaded regions range from the 5^th^ through 95^th^ percentiles of each measure over the area traveled by study participants on that day. Max maximum, min minimum.

The distributions of LST, maximum air temperature, and minimum air temperature over time within a 200 m buffer of the area traveled by all of the study participants (regardless of time elapsed therein) appear in Table 1 and Fig. 1. The average LST over this area during the study period ranged from a low of 286 K to a high of 319 K, with an average within-day spatial variation (standard deviation) of 8.36 K. Maximum daily air temperature ranged over time from an average (over pixels on that day) of 282 K to 314 K, and the corresponding average minimum daily air temperature ranged from 273 K to 295 K. Within-day spatial variability was lower for air temperature (average standard deviation of 3.75 K and 3.02 K, respectively for maximum and minimum) than for LST (8.36 K).

### Assessment of heat exposure

#### Mobility-based

We calculated two mobility-based measures of heat exposure for both LST and air temperature: a time-weighted mean (denoted as ${{mean}}_{{mobility}-{based}}$ to facilitate notation below) and the highest average value of 10 min intervals of their activity paths (${{maximum}}_{{mobility}-{based}}$). We calculated the time-weighted mean as a measure of cumulative exposure and the maximum over 10 min intervals as a measure of the highest acute exposure.

##### Mean

To calculate mean mobility-based exposure to LST, we began by extracting LST values at the point location of each GPS ping for each participant, using both the image that most closely preceded the date of the GPS ping (the index date) and the image that most closely followed that day. For example, if a participant recorded activity on March 15^th^, 2015, we extracted the LST value from March 9^th^, 2015, and March 25^th^, 2015 for each ping recorded on March 15^th^, 2015. Then, for each ping on each index date, we calculated the weighted average of its two LST values, weighting the two values by the inverse of the time between the index date and the image date so that the LST of the image nearer in time would receive a stronger weight in the average. Specifically, if ${{LST}}_{{mean}}$ is the weighted average between these two days, then ${{LST}}_{{mean}}=\frac{{{LST}}_{{earlier}}* {{wt}}_{{earlier}}+{{LST}}_{{later}}* {{wt}}_{{later}}}{{{wt}}_{{earlier}}+{{wt}}_{{later}}}$, where ${{wt}}_{{earlier}}=\frac{{number\; of\; days\; between\; the\; two\; LST\; images}}{{index\; date}-{date\; of\; earlier\; LST\; image}}$ and ${{{wt}}_{{later}}=\frac{{number\; of\; days\; between\; the\; two\; LST\; images}}{{date\; of\; later\; LST\; image}-{index\; date}}}$. For each participant, we then calculated their weighted average mobility-based LST exposure, weighting the value of each point’s value by its elapsed time until the next one, including overnight time.

To calculate mean mobility-based exposure to air temperature, we broadly followed the same method with some differences because air-temperature is available at a daily temporal resolution. To roughly estimate minute-level air-temperature exposure within day, we averaged the maximum and minimum air temperature values at the location of each GPS ping based on the ping’s time of day, assuming the maximum air temperature occurred at 3 pm and that the minimum air temperature occurred at that day’s sunrise. We calculated sunrise time for all days during the study period for San Diego, California using NOAA’s calculator [36]. Specifically, again taking an inverse-distance-in-time-weighting approach, if ${{AT}}_{{mean}}$ denotes the weighted average between the maximum and minimum air temperatures at a time of day (the index time) on the index date, then ${{AT}}_{{mean}}=\frac{{{AT}}_{\max }* {{wt}}_{\max }+{{AT}}_{\min }* {{wt}}_{\min }}{{{wt}}_{\max }+{{wt}}_{\min }}$, where ${{{wt}}_{\max }=\frac{{{\rm{Time}}} \; {{\rm{until}}}\; {{\rm{or}}}\; {{\rm{after}}}3{{\rm{pm}}}+{{\rm{Time}}}\; {{\rm{since}}}\; {{\rm{or}}}\; {{\rm{until}}}\; {{\rm{sunrise}}}}{{{\rm{Time}}}\; {{\rm{until}}}\; {{\rm{or}}}\; {{\rm{after}}}3{{\rm{pm}}}}}$, and ${{{wt}}_{\min }=\frac{{{\rm{Time}}}\; {{\rm{until}}}\; or\; {{\rm{after}}}3{{\rm{pm}}}+{{\rm{Time}}}\; {{\rm{since}}}\; {{\rm{or}}}\; {{\rm{until}}}\; {{\rm{sunrise}}}}{{{\rm{Time}}}\; {{\rm{since}}}\; {{\rm{or}}}\; {{\rm{until}}}\; {{\rm{sunrise}}}}}$. Time until or after 3 pm is the elapsed time until 3 pm if the index time is before 3 pm or the elapsed time after 3 pm if after. The time since or until sunrise is the elapsed since sunrise if the index is before 3 pm or until sunrise if after 3 pm.

We then calculated each participant’s mobility-based mean air-temperature exposure as the weighted average of these estimated air-temperature values over their duration of GPS follow-up, weighting each GPS ping’s air temperature by the elapsed time until the next GPS ping, again allowing that the elapsed time until the next GPS ping may include non-wear time between going to bed and waking the next day.

To assess the impact of our decision to include non-wear time in the time-weighted exposure measures, we also calculated the weighted mean LST and air temperature excluding non-wear time.

##### Maximum

To assess acute exposure to the heat indicators, we grouped participant’s GPS points into ordered sequences of 10 min intervals and took the mean exposure value of each 10 min interval over their constituent point-level values. We grouped points into 10 min intervals because heat stroke can develop in 10 min [24]. We then found the maximum (denoted as ${{maximum}}_{{mobility}-{based}}$) of each participant’s 10 min-interval averages.

#### Residence-based

##### Mean

To assess residence-based exposure to these heat indicators (${{mean}}_{{residence}-{based}}$), we first defined participants’ home location. To define their home location, we drew a grid of hexagons over San Diego County, each with an area of 1000 m^2^ (each side ~ 62 m). We defined home as the centroid of the hexagon where the participant spent the most elapsed time, where elapsed time is defined by the difference between sequential GPS pings, including the difference between the last ping of one day and the first of the next. We inferred home location as the location where the participant spent the most time because we observed that many study participants were rarely at the home address that they provided in the survey. Inferring home location based on elapsed time is consistent with research using mobile-phone-based location data that has inferred home location as the location where their device spent the most time overnight [37–39]. For 587 (98%) of the 599 participants, the hexagon where the participant spent the most elapsed time overnight (8 p.m.—6 a.m.) was either the same as (n = 578) or shared an adjacent edge with (*n* = 9) the hexagon where they spent the most total elapsed time. We chose to use the hexagon where the most total elapsed time was spent rather than that where the most time was spent at night because for some of the 2% (*n* = 12) of participants for which the two locations differed, the hexagon where the most time was spent at night was either a place of employment (suggesting night-shift work) or a campground (suggesting the study period may have coincided with a vacation).

To calculate the mean residence-based exposure to each indicator for each participant, we followed the same approach as for mobility-based means, but instead of using the actual location of each GPS ping, we extracted the heat-indicator value corresponding to the participant’s home location at the time of each GPS ping. We then summarized the information the same way as above.

##### Maximum

The approach for calculating maximum residence-based exposure (${{maximum}}_{{residence}-{based}}$) for LST differed from that of air temperature because of the differences in the temporal resolution of these indicators. Each participant’s maximum residence-based LST exposure is the maximum of their day-level residence-based LST exposure during their days of GPS follow-up. In contrast, the maximum residence-based exposure to air temperature is the maximum value of each participant’s set of 10 min intervals, where the location used to calculate the interval-specific means is always their home location.

### Analysis

We compared mobility-based means with residence-based means and mobility-based maximums with residence-based maximums using differences: ${{difference}}_{{means}}={{mean}}_{{mobility}-{based}}-{{mean}}_{{residence}-{based}}$; ${{difference}}_{{maximums}}={{maximum}}_{{mobility}-{based}}-{{maximum}}_{{residence}-{based}}$. We also compared means using Pearson correlations.

To explore variation in the comparison measures by mobility patterns and socio-demographic characteristics, we stratified results by tertiles of average daily distance traveled (5.63–41.5 km; 41.5–65 km; 65–368 km), age (35–50 years old; 51–65 years old; 66–80 years old), sex (female; male), income (<$30 k; $30 k–$55 k; $55k + ), and race and ethnicity (white; Latino; Asian; Black; Native American or Pacific Islander).

We plotted histograms to visually assess the distribution of the difference measures.

## Results

### Description of mobility patterns

Socio-demographic characteristics of the sample appear in Table 2 and have been reported previously [20]. Table 2 also presents the average daily distance traveled and the percent of time spent within 200 meters of inferred home. On average, study participants traveled a mean of 59 km daily and spent 74% of their GPS follow-up time at home, including non-wear time. There was considerable variability in daily distance traveled between (standard deviation (SD) between individuals=34 km) and within (SD = 44 km) individuals. Older adults aged 66-80 years tended to travel less daily distance (mean=50 km) and spend more time at home (mean=79%) than their younger counterparts. Higher-income individuals (above $55,000) traveled more daily distance (mean=66 km) than those in the other income categories, and Latino individuals traveled more daily distance (mean=64 km) than individuals of other races and ethnicities.

**Table 2:** Daily distance traveled and percent of time spent near home among study population, stratified by socio-demographic characteristics.

		Daily distance traveled (km)			Percent of time spent within 200 m of home	
Characteristic	*n* (%)	Mean	Between-individual SD	Mean of within-individual SDs	Mean	Between-individual SD
**Overall**	599	59	34	44	74%	14%
**Age (years), mean (SD)**	59 (11)					
35–50 years old	141 (24%)	68	46	51	71%	13%
51–65 years old	269 (45%)	61	30	45	73%	14%
66–80 years old	189 (32%)	50	27	39	79%	13%
**Sex**						
Female	335 (56%)	57	33	42	74%	14%
Male	264 (44%)	61	35	47	74%	14%
**Income**						
Income <$30k	162 (27%)	50	29	36	76%	14%
Income $30k-$55k	136 (23%)	57	31	44	73%	15%
Income $55k+	278 (46%)	66	38	50	73%	13%
Missing	23 (4%)	51	24	39	77%	14%
**Race/ethnicity**						
White	296 (49%)	55	34	41	76%	13%
Latino	250 (42%)	64	34	48	72%	14%
Asian	18 (3%)	58	24	35	68%	13%
Black	17 (3%)	59	30	67	79%	13%
Native American or Pacific Islander	13 (2%)	58	52	39	74%	18%
Missing	5 (1%)	43	29	27	73%	21%

*SD* Standard deviation.

eFigure 1 maps the home census tracts of the study participants, and eFigure 2 maps where participants spent the most time, classifying census tracts by the proportion of the total person-time spent in them over all participants. Most participants lived in and traveled through the urbanized west and southwest regions of San Diego County. A small share of their time was spent in the more rural central and eastern areas of the country.

### Exposure measures

**Fig. 2:**
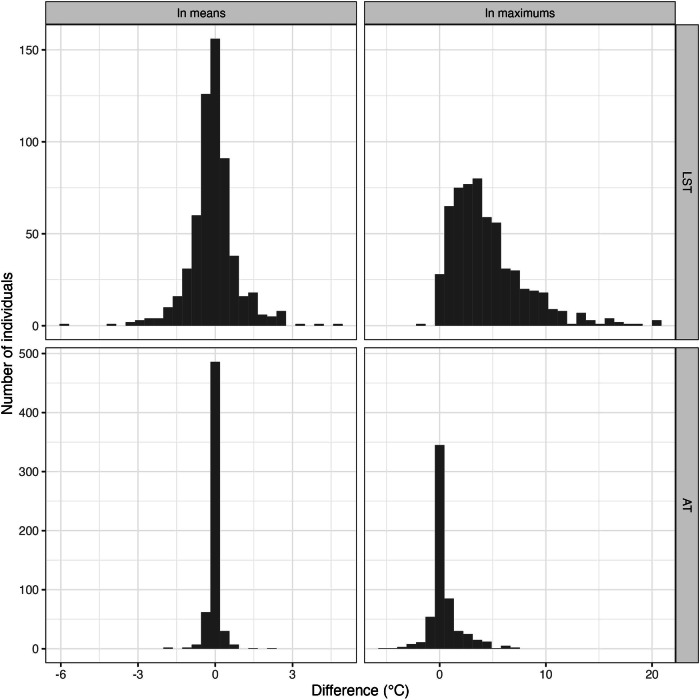
Histograms of individual-level differences between means and maximums of each measurement method for each heat indicator. These difference measures are defined in the analysis section of the methods text. LST land surface temperature, AT air temperature, °C degrees Celsius.

The overall mobility-based mean LST exposure (34.7 degrees Celsius [°C]; Table 3) was almost equivalent to the corresponding residence-based mean (34.8 °C; mean difference in means = −0.09 °C; Pearson correlation of means=0.993; Table 4). There was some between-individual variability in this difference measure (standard deviation [SD] of difference=0.94 °C; Table 4), but the distribution was concentrated around 0 (Fig. 2). The mean difference between the overall mobility-based mean air temperature exposure (19.2 °C) and the corresponding residence-based mean (19.2 °C) was even smaller (−0.02 °C), as was its between-individual variability (SD of difference=0.24 °C).

**Table 3:** Summary measures (mean and maximum) of mobility-based (M) and residence-based (R) exposure to heat indicators overall, overall excluding non-wear time, and by daily distance traveled and socio-demographic characteristics.

			Within-individual mean (°C)		Within-individual maximum (°C)	
Heat indicator	Characteristic^a^	Method (M or R)	Mean	SD	Mean	SD
Land Surface Temperature	All individuals	M	34.7	7.5	40.3	8.2
		R	34.8	7.7	35.8	7.6
	All individuals; non-wear time excluded	M	34.6	7.3	40.3	8.2
		R	34.8	7.7	35.8	7.6
Air temperature	All individuals	M	19.2	3.5	28.6	4.4
		R	19.2	3.5	28.2	4.3
	All individuals; non-wear time excluded	M	19.8	3.5	28.6	4.4
		R	19.8	3.5	28.2	4.3
	**Daily distance traveled (km)**					
Land Surface Temperature	[5.63, 41.5]	M	34.8	6.9	39.3	7.3
		R	34.8	7.0	35.7	7.0
	(41.5, 65]	M	34.8	7.6	40.6	8.4
		R	34.9	7.8	35.9	7.8
	(65, 368]	M	34.6	7.8	40.9	8.7
		R	34.7	8.2	35.7	8.1
Air temperature	[5.63, 41.5]	M	19.1	3.7	28.0	4.2
		R	19.1	3.7	27.7	4.2
	(41.5, 65]	M	19.3	3.4	28.7	4.3
		R	19.3	3.5	28.3	4.3
	(65, 368]	M	19.1	3.4	29.2	4.6
		R	19.2	3.4	28.8	4.5
	**Age category**					
Land Surface Temperature	(35,50]	M	34.7	7.0	39.8	7.9
		R	34.9	7.2	35.9	7.2
	(50,65]	M	35.4	7.9	40.8	8.5
		R	35.5	8.1	36.5	8.0
	(65,80]	M	33.8	7.1	39.9	8.0
		R	33.7	7.3	34.7	7.3
Air temperature	(35,50]	M	19.3	3.9	29.0	4.9
		R	19.3	3.9	28.8	4.7
	(50,65]	M	19.2	3.4	28.8	4.2
		R	19.2	3.5	28.4	4.2
	(65,80]	M	19.0	3.3	28.1	4.2
		R	19.0	3.3	27.6	4.1
	**Sex**					
Land Surface Temperature	Women	M	35.2	7.2	40.6	7.8
		R	35.2	7.3	36.2	7.3
	Men	M	34.1	7.7	39.9	8.6
		R	34.3	8.1	35.2	8.0
Air temperature	Women	M	19.2	3.3	28.7	4.3
		R	19.3	3.3	28.2	4.3
	Men	M	19.0	3.8	28.6	4.5
		R	19.1	3.8	28.3	4.4
	**Income category**					
Land Surface Temperature	< $30k	M	36.8	6.8	42.0	7.6
		R	36.9	6.9	37.9	6.8
	$30k–$55k	M	35.6	7.0	40.4	7.9
		R	35.8	7.1	36.9	7.2
	$55k+	M	33.1	7.7	39.3	8.6
		R	33.1	8.0	34.1	7.9
	Missing	M	34.2	6.8	39.1	8.1
		R	34.2	7.1	35.2	7.5
Air temperature	<$30k	M	19.4	3.7	28.6	4.6
		R	19.4	3.7	28.1	4.4
	$30k–$55k	M	19.2	3.6	28.7	4.4
		R	19.2	3.7	28.4	4.5
	$55k+	M	19.0	3.4	28.6	4.3
		R	19.0	3.4	28.2	4.2
	Missing	M	18.7	3.0	28.4	4.4
		R	18.7	3.1	28.3	4.4
	**Race or ethnicity**					
Land Surface Temperature	White	M	33.0	7.5	38.7	8.5
		R	33.0	7.7	33.9	7.7
	Hispanic or Latino	M	36.8	7.0	42.2	7.7
		R	37.1	7.2	38.1	7.0
	Asian	M	31.7	7.1	39.1	8.2
		R	31.5	7.2	32.7	7.2
	Black	M	36.1	6.9	40.8	6.9
		R	36.4	6.8	37.5	6.5
	Native American or Pacific Islander	M	36.3	6.2	41.3	6.1
		R	36.8	6.3	38.0	5.9
	Missing	M	32.6	7.3	37.8	7.9
		R	32.9	7.3	33.9	6.7
Air temperature	White	M	18.7	3.4	28.4	4.5
		R	18.7	3.4	27.9	4.3
	Hispanic or Latino	M	19.5	3.4	28.8	4.1
		R	19.6	3.4	28.4	4.1
	Asian	M	19.5	5.1	28.3	5.3
		R	19.6	5.1	28.4	5.5
	Black	M	20.9	3.4	30.8	5.6
		R	21.0	3.4	30.4	6.0
	Native American or Pacific Islander	M	19.3	4.1	30.2	4.1
		R	19.3	4.2	29.4	4.7
	Missing	M	17.6	2.6	27.3	3.1
		R	17.5	2.6	27.5	3.1

^a^Unless otherwise specified, applicable weighted means include non-wear time.

*°C* degrees Celsius, *M* Mobility-based method, *R* Residence-based method, *SD* standard deviation.

**Table 4:** Measures comparing mobility-based with residence-based summary measures of exposure to heat indicators overall, overall excluding non-wear time, and by daily distance traveled and socio-demographic characteristics.

		Difference in within-individual means (°C)			Difference in within-individual maximums (°C)	
Heat indicator	Characteristic^a^	Mean	SD	Pearson correlation of means	Mean	SD
Land Surface Temperature	All individuals	−0.09	0.94	0.993	4.51	3.53
	All individuals; non-wear time excluded	−0.17	1.41	0.984	4.51	3.53
Air temperature	All individuals	−0.02	0.24	0.998	0.40	1.41
	All individuals; non-wear time excluded	−0.02	0.35	0.995	0.40	1.41
	**Daily distance traveled (km)**					
Land Surface Temperature	[5.63,41.5]	0.01	0.75	0.994	3.62	2.73
	(41.5,65]	−0.14	0.90	0.994	4.70	3.40
	(65,368]	−0.13	1.14	0.991	5.20	4.15
Air temperature	[5.63,41.5]	−0.01	0.11	1.000	0.32	1.01
	(41.5,65]	−0.01	0.28	0.997	0.44	1.44
	(65,368]	−0.05	0.30	0.996	0.44	1.70
	**Age category**					
Land Surface Temperature	(35,50]	−0.20	0.97	0.991	3.91	3.29
	(50,65]	−0.17	0.97	0.993	4.32	3.16
	(65,80]	0.11	0.86	0.993	5.22	4.07
Air temperature	(35,50]	−0.02	0.17	0.999	0.23	1.34
	(50,65]	−0.05	0.28	0.997	0.43	1.57
	(65,80]	0.00	0.24	0.997	0.49	1.20
	**Sex**					
Land Surface Temperature	Women	−0.08	0.80	0.994	4.37	3.25
	Men	−0.10	1.10	0.991	4.68	3.86
Air temperature	Women	−0.02	0.25	0.997	0.50	1.42
	Men	−0.02	0.24	0.998	0.28	1.39
	**Income category**					
Land Surface Temperature	< $30k	−0.10	0.83	0.993	4.08	3.63
	$30k–$55k	−0.22	1.02	0.990	3.59	3.09
	$55k+	−0.02	0.93	0.994	5.25	3.57
	Missing	−0.06	1.27	0.984	3.96	3.09
Air temperature	<$30 k	0.01	0.28	0.997	0.54	1.40
	$30k–$55k	−0.04	0.20	0.998	0.28	1.42
	$55k+	−0.03	0.24	0.997	0.41	1.46
	Missing	0.00	0.15	0.999	0.05	0.50
	**Race or ethnicity**					
Land Surface Temperature	White	0.03	0.89	0.994	4.82	3.69
	Latino	−0.21	0.93	0.992	4.16	3.41
	Asian	0.14	1.55	0.977	6.34	3.83
	Black	−0.25	1.15	0.986	3.30	1.77
	Native American or Pacific Islander	−0.50	0.69	0.994	3.33	2.36
	Unknown	−0.29	0.86	0.993	3.94	2.43
Air temperature	White	0.00	0.23	0.998	0.46	1.29
	Latino	−0.05	0.27	0.997	0.37	1.58
	Asian	−0.06	0.08	1.000	−0.19	0.84
	Black	−0.07	0.19	0.999	0.38	1.32
	Native American or Pacific Islander	−0.03	0.13	0.999	0.74	1.40
	Unknown	0.13	0.21	0.997	−0.18	0.44

^a^Unless otherwise specified, applicable weighted means include non-wear time.

*°C* Degrees Celsius, *SD* Standard deviation.

When non-wear time was excluded from the mobility-based weighted mean, the difference between the two means remained negligible though was slightly greater for LST (−0.17 °C) and remained the same for air temperature (−0.02 °C).

Meaningful differences emerged, however, when comparing maximums, particularly for LST. The mean mobility-based maximum was 40.3 °C (SD = 8.2 °C) compared with a mean residence-based maximum of 35.8 °C (SD = 7.6 °C), a difference of 4.51 °C (SD = 3.53 °C). The difference in maximums was considerably smaller for air temperature (mean=0.40 °C; SD = 1.41 °C) but nevertheless greater than the corresponding difference in means.

Although differences in means were uniformly small (all less than 0.3 °C) when stratified by daily distance traveled and socio-demographic characteristics, differences were slightly larger in some groups (Table 4). For example, among those who traveled 65−368 km daily, the mean difference in means was −0.13 °C for LST and −0.05 °C for air temperature compared with 0.01 °C and −0.01 °C, respectively, among those who traveled 5.63−41.5 daily km. Relative to other groups, differences in means were also slightly larger among younger adults (e.g., −0.20 °C for LST) and non-white individuals. For example, white individuals had a mean difference in means of 0.03 °C for LST and 0.00 °C for air temperature compared with −0.25 °C and −0.07 °C, respectively for Black individuals. These relative patterns roughly held for the differences in maximums.

## Discussion

This study compared a mobility-based approach for estimating exposure to heat indicators—land surface temperature and air temperature—with a residence-based approach in a San Diego study population. Exposure measures were almost equivalent between the two methods when comparing means, with some heterogeneity when stratified by daily distance traveled and sociodemographic characteristics. This is a somewhat unusual finding in the context of much of the activity-space literature that, speaking generally, typically observes that exposure measures considering activity space differ from those that only consider place of residence.

Differences between the two approaches were more considerable when comparing the maximum exposure measured over the course of each participant’s GPS follow-up, especially for LST, for which the mean difference in maximums was 4.51 °C, as compared with 0.4 °C for air temperature. This result can likely be explained by the differences in spatial and temporal resolution between the two measures. LST has high spatial resolution (30 m) but poor temporal resolution (every 15 days at best), whereas air temperature has poorer spatial resolution (4 km) and higher temporal resolution (daily). The principal difference between the mobility-based and residence-based methods is that the mobility-based method considers both the spatial and temporal variation of the participant’s day-to-day travel patterns, whereas the residence-based approach does not consider spatial variation in the travel patterns but does consider the underlying temporal variation of the measure at the home location. It follows, then, that the residence-based approach would capture more total variation for the measure with higher temporal resolution (air temperature), given participants spent much of their time at home, whereas the residence-based approach would capture less of the total variation for the measure with worse temporal resolution but better spatial resolution.

Although the activity-space literature has become vast, there have not been many studies that have examined measures of microclimate indicators, specifically, using an activity-space-based approach. One study in China used mobile-phone-derived location data to dynamically assess exposure to green space [40]. That study did not compare mobility-based exposure assessment with residence-based exposure assessment, however. In the study comparing residence-based with mobility-based measurement of land surface temperature in Dhaka, Bangladesh [27], the authors found, in contrast with our results, that particularly among people who commuted between the suburbs and the city center, the residence-based measurement underestimated land surface temperature exposure compared with the activity space-based measure by a difference of 2.4 °C. In agreement with our results, other mobility groups in their study had less of a difference, however. For example, the difference in land surface temperature was −0.03 °C for the group who lived within Dhaka Metropolitan Area and remained there all day and was −0.08 °C for those who lived in the suburbs and remained there all day. These values are about the same as our overall difference in means of −0.09 °C. This result follows in that the place of residence will more accurately reflect mobility-based activity patterns if the person remains at or near the home for most of the day. Our study is broadly in agreement with research from western New York, US, which also found that residence-based and mobility-based measures of exposure to green space agreed with one another, with some differences by personal characteristics [41].

One reason that residence-based and mobility-based means generally agree in our study may be the fact that we weighted mobility-based means proportionally to elapsed time, including elapsed time overnight. In this way, our approach is similar to methods described by Morrison et al. [16], Raskind et al. [18], and Jankowska et al. [31]. Unlike some of these approaches [31], however, our method here preserves the unit of the exposure measure, which is important for making interpretable comparisons in this analysis and for comparing our results to other studies.

Whether and by how much to weight the home location in measuring mobility-based exposure is a common discussion topic in the activity-space literature [15, 16, 31, 42], and the answer, almost always, is that it depends on the research question under study and specifically the hypothesized mechanism through which the measured exposure affects health. In this study, we included overnight time in our main analysis because extreme heat exposure can be particularly harmful at night, especially for older adults who comprised a large share of our study population (32% 66 years and older) [26, 43–45]. Nighttime heat may specifically have adverse consequences in San Diego, where air conditioning is uncommon [46]. Nighttime heat events are expected to become more frequent in California as the climate warms [47]. When we excluded non-wear time from the mobility-based measures, differences between means increased slightly but remained small (Table 4).

Our results also highlight, however, that if transient exposure to heat is of interest, then the residence-based maximum exposure may differ from the maximum value experienced during day-to-day travel. Whether transient or cumulative exposure to heat is most relevant will depend on the health outcome [1].

Our study has limitations that should be considered. Research has shown that LST does not necessarily correspond with human thermal comfort [10], and other measures such as mean radiant temperature may better reflect human heat exposure. Mean radiant temperature was unfortunately not available at the needed spatial extent and resolution for this study. To address the limitation of LST, we also included air temperature in our analysis. While air temperature affects thermal comfort more directly than LST [5], the air-temperature measure used in this analysis does not consider radiation. It can thus be interpreted as the neighborhood-scale air temperature as though people were in the shade. The spatial resolution of air temperature was coarser than that of LST but was fine enough to allow for spatial variation within the course of most participants’ day-to-day travel patterns, as participants traveled on average 59 km (SD = 34 km) per day. On a 59 km trip, a participant could plausibly travel through 14 air-temperature gridded pixels. It is also important to note that our goal was to measure the heat-related conditions of the outdoor environment, whether participants were outdoors or not. Participants may have been indoors or traveling in cars during much of their travel patterns. If they had air conditioning available while indoors, they would of course not be as affected by the outdoor heat conditions. We nevertheless find the question of the temperature of the outdoor environment to be relevant, as air conditioning is uncommon in San Diego [46] and is not always available in cars.

In summary, mobility-based measures of heat exposure were not appreciably different from those considering place of residence alone, although differences were slightly greater in certain socio-demographic groups. When considering the maximum value experienced, values differed more strongly between mobility-based and residence-based methods, especially for LST. This result could be meaningful for health pathways between transient heat exposure and acute health outcomes. Future studies may wish to assess mobility-based and residence-based measures using other measures that more directly align with human thermal burden such as mean radiant temperature as data availability permits.

## Supplementary information

Supplementary Material

## Data Availability

The data on study participants are not available, as the data include identifiable information such as day-to-day travel patterns. We have, however, posted code that prepares the Landsat data on land-surface temperature and the GridMET data on air temperature, as these data are publicly available. That code can be found here: https://github.com/michaeldgarber/microclim-static-v-dynam/tree/main/scripts.
