# Supplementary material for: Is home where the heat is? comparing residence-based with mobility-based measures of heat exposure in San Diego, California
Source: J Expo Sci Environ Epidemiol. 2024 Sep 11;35(5):802–12. doi: 10.1038/s41370-024-00715-5 (PMC12318250; doi:10.1038/s41370-024-00715-5)
Supplement: Supplementary file 1 — Supplementary Material [file 41370_2024_715_MOESM1_ESM.docx]

***Supplement to***

**Is home where the heat is? comparing residence-based with mobility-based measures of heat exposure in San Diego, California**

Revised May 6, 2024

Table of Contents

[eFigure 1: Home census tracts 2](#_Toc165379545)

[eFigure 2: Map of total time spent (hours) in census tracts by participants. 3](#_Toc165379546)

[eTable 1. LandSat images used in analysis 4](#_Toc165379547)

# eFigure 1: Home census tracts


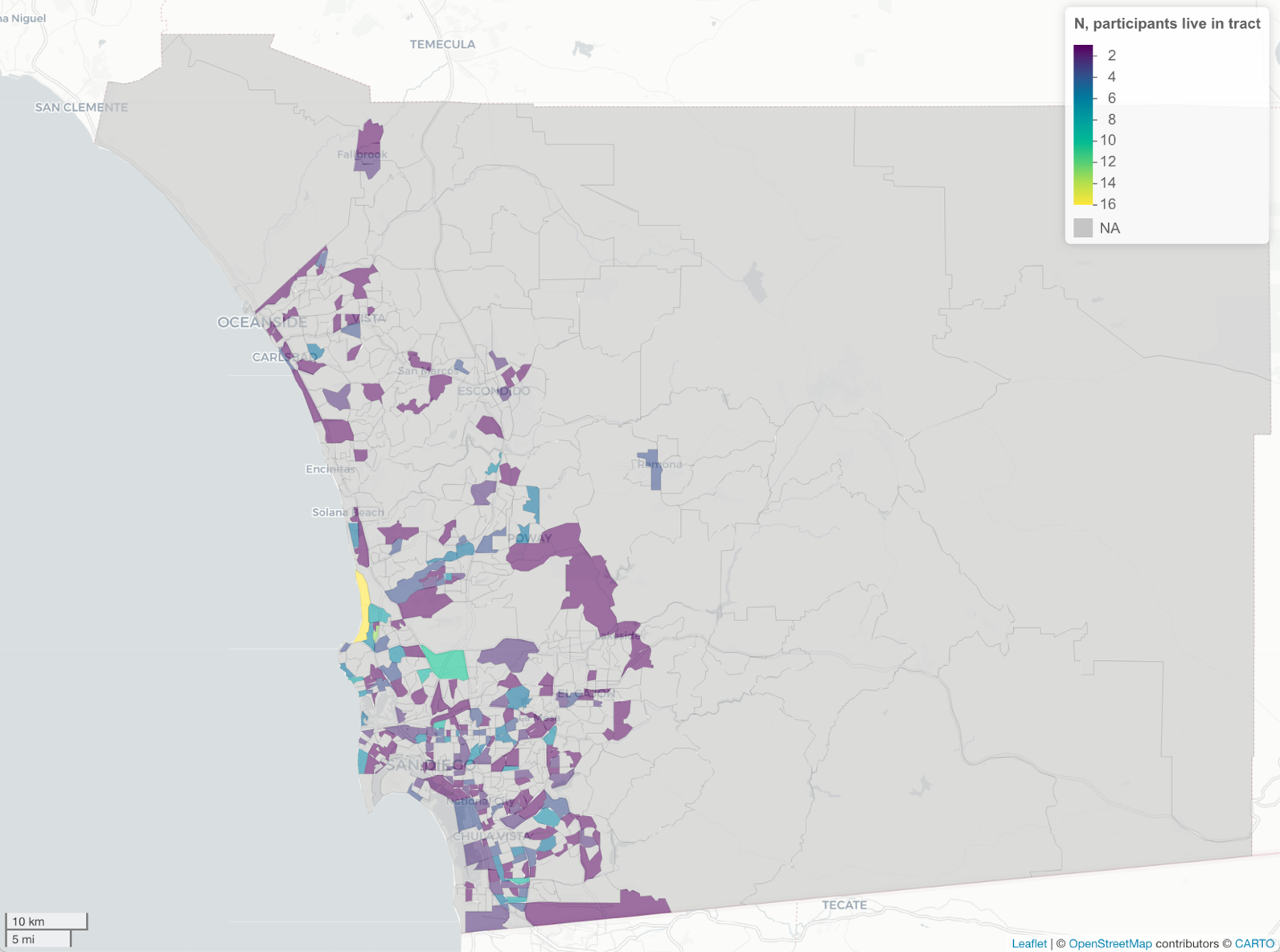


**eFigure 1.** Map of home census tracts of study participants, where home census tract is defined as that in which the participant spent the most time.

# eFigure 2: Map of share of total person-time spent in census tracts by all participants.


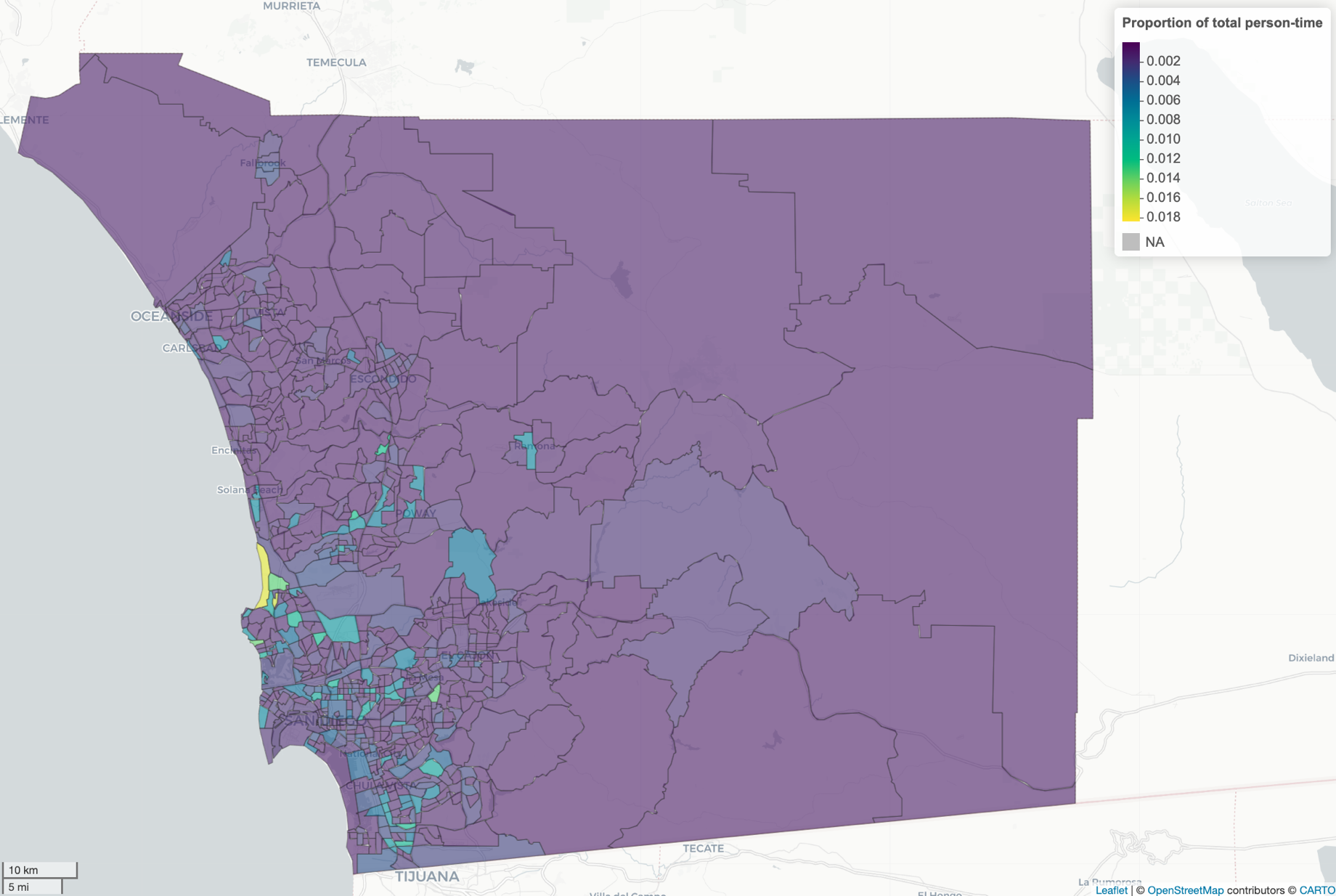


**eFigure 2.** Map of share of total person-time spent in census tracts by all participants.

# **eTable 1**. LandSat images used in analysis

| **eTable 1**. LandSat images used in analysis |  |  |  |
| --- | --- | --- | --- |
| Image identifier | Image date | Measure | Percent land cloud cover |
| LC08_L2SP_040037_20140930_20200910_02_T1_ST_B10.TIF | 2014-09-30 | ST_B10 | 0.03 |
| LC08_L2SP_040037_20141117_20200910_02_T1_ST_B10.TIF | 2014-11-17 | ST_B10 | 0.05 |
| LC08_L2SP_040037_20141219_20200910_02_T1_ST_B10.TIF | 2014-12-19 | ST_B10 | 0.18 |
| LC08_L2SP_040037_20150104_20200910_02_T1_ST_B10.TIF | 2015-01-04 | ST_B10 | 2.16 |
| LC08_L2SP_040037_20150205_20200909_02_T1_ST_B10.TIF | 2015-02-05 | ST_B10 | 0.85 |
| LC08_L2SP_040037_20150309_20200909_02_T1_ST_B10.TIF | 2015-03-09 | ST_B10 | 0.19 |
| LC08_L2SP_040037_20150325_20200909_02_T1_ST_B10.TIF | 2015-03-25 | ST_B10 | 0.08 |
| LC08_L2SP_040037_20150512_20200909_02_T1_ST_B10.TIF | 2015-05-12 | ST_B10 | 2.62 |
| LC08_L2SP_040037_20150715_20200908_02_T1_ST_B10.TIF | 2015-07-15 | ST_B10 | 0.04 |
| LC08_L2SP_040037_20150816_20200909_02_T1_ST_B10.TIF | 2015-08-16 | ST_B10 | 0.35 |
| LC08_L2SP_040037_20150917_20200908_02_T1_ST_B10.TIF | 2015-09-17 | ST_B10 | 0.46 |
| LC08_L2SP_040037_20151003_20200908_02_T1_ST_B10.TIF | 2015-10-03 | ST_B10 | 1.39 |
| LC08_L2SP_040037_20151120_20200908_02_T1_ST_B10.TIF | 2015-11-20 | ST_B10 | 0.13 |
| LC08_L2SP_040037_20160208_20200907_02_T1_ST_B10.TIF | 2016-02-08 | ST_B10 | 0.17 |
| LC08_L2SP_040037_20160224_20200907_02_T1_ST_B10.TIF | 2016-02-24 | ST_B10 | 0.11 |
| LC08_L2SP_040037_20160327_20200907_02_T1_ST_B10.TIF | 2016-03-27 | ST_B10 | 2.46 |
| LC08_L2SP_040037_20160717_20200906_02_T1_ST_B10.TIF | 2016-07-17 | ST_B10 | 0.47 |
| LC08_L2SP_040037_20160802_20200906_02_T1_ST_B10.TIF | 2016-08-02 | ST_B10 | 0.59 |
| LC08_L2SP_040037_20160903_20200906_02_T1_ST_B10.TIF | 2016-09-03 | ST_B10 | 0.65 |
| LC08_L2SP_040037_20161021_20200905_02_T1_ST_B10.TIF | 2016-10-21 | ST_B10 | 0.03 |
| LC08_L2SP_040037_20161106_20200905_02_T1_ST_B10.TIF | 2016-11-06 | ST_B10 | 0.98 |
| LC08_L2SP_040037_20161122_20200905_02_T1_ST_B10.TIF | 2016-11-22 | ST_B10 | 0.17 |
| LC08_L2SP_040037_20161208_20200905_02_T1_ST_B10.TIF | 2016-12-08 | ST_B10 | 0.24 |
| LC08_L2SP_040037_20170125_20200905_02_T1_ST_B10.TIF | 2017-01-25 | ST_B10 | 2.06 |
| LC08_L2SP_040037_20170314_20200904_02_T1_ST_B10.TIF | 2017-03-14 | ST_B10 | 0.38 |
| LC08_L2SP_040037_20170330_20200904_02_T1_ST_B10.TIF | 2017-03-30 | ST_B10 | 0.54 |
| LC08_L2SP_040037_20170415_20200904_02_T1_ST_B10.TIF | 2017-04-15 | ST_B10 | 0.10 |
| LC08_L2SP_040037_20170501_20200904_02_T1_ST_B10.TIF | 2017-05-01 | ST_B10 | 0.04 |
| LC08_L2SP_040037_20170602_20200903_02_T1_ST_B10.TIF | 2017-06-02 | ST_B10 | 0.92 |
| LC08_L2SP_040037_20170618_20200903_02_T1_ST_B10.TIF | 2017-06-18 | ST_B10 | 0.64 |
| LC08_L2SP_040037_20170704_20200903_02_T1_ST_B10.TIF | 2017-07-04 | ST_B10 | 0.24 |
| LC08_L2SP_040037_20170805_20200903_02_T1_ST_B10.TIF | 2017-08-05 | ST_B10 | 0.51 |
| LC08_L2SP_040037_20170821_20201015_02_T1_ST_B10.TIF | 2017-08-21 | ST_B10 | 0.04 |
| LC08_L2SP_040037_20170906_20200903_02_T1_ST_B10.TIF | 2017-09-06 | ST_B10 | 2.55 |
| LC08_L2SP_040037_20171008_20200903_02_T1_ST_B10.TIF | 2017-10-08 | ST_B10 | 0.89 |
